# External validation and optimization of the Limoges score to improve the delayed bleeding risk prediction after colorectal endoscopic submucosal dissection: the Delayed Bleeding ESD (DEBE) score

**DOI:** 10.1055/a-2814-4950

**Published:** 2026-03-23

**Authors:** Eduardo Albéniz, Sheyla Montori, Mónica Enguita-Germán, José C. Marín-Gabriel, Alberto Herreros de Tejada, Timothée Wallenhorst, Jérôme Rivory, Romain Legros, Thibault Degand, Jean-Baptiste Chevaux, Felipe Ramos-Zabala, Yann Le Baleur, Florian Rostain, Arthur Berger, Pedro J. Rosón, Álvaro Terán, Maximilien Barret, Philipe Leclercq, Edouard Chabrun, Marion Schaeffer, Bertrand Brieau, Safia Boukechiche, Gonzalo Hijos-Mallada, Mathieu Pioche, Jérémie Jacques, on behalf of the Colorectal ESD Consortium Working Group, Alexandru Lupu, José Santiago García, Hugo Lepetit, Hugo Uchima, Francisco J. Gallego Rojo, Joaquín de la Peña García, Jérémie Albouys, Paul Bonniaud, Stanislas Chaussade, Joaquín Rodríguez-Sánchez, Julien Geay, Ana Amorós Tenorio, Arthur Belle, Martin Dahan, Diana M. García Romero, Enrique Rodriguez de Santiago, Sofía Parejo-Carbonell, Óscar Nogales, Olivier Plomteux, Jean-Baptiste Zeevaert, Gloria Fernández-Esparrach, Andrés Sánchez-Yagüe, Unai Goikoetxea, Thomas Grainville, Fermín Estremera-Arevalo, Marta Gómez Alonso

**Affiliations:** 1 Endoscopy Unit, Gastroenterology Department, Hospital Universitario de Navarra (HUN), Pamplona, Spain; 2 Gastroenterology Research Unit, Navarrabiomed, Pamplona, Spain; 3 Digestive System and Metabolism Diseases, Instituto de Investigación Sanitaria de Navarra (IdiSNA), Pamplona, Spain; 4 Universidad Pública de Navarra (UPNA), Pamplona, Spain; 5 Department of Methodology, Navarrabiomed, Pamplona, Spain; 6 Universidad Pública de Navarra (UPNA), Pamplona, Spain; 7 Primary Care, Healthcare, and Health Services, Instituto de Investigación Sanitaria de Navarra (IdiSNA), Pamplona, Spain; 8 Hospital Universitario de Navarra (HUN), Pamplona, Spain; 9 Endoscopy Unit, Department of Gastroenterology, Hospital Universitario 12 de Octubre, Madrid, Spain; 10 i+12 Research Institute, Madrid, Spain; 11 Universidad Complutense de Madrid, Madrid, Spain; 12 Department of Digestive Diseases, Hospital Universitario Puerta de Hierro-Majadahonda, Madrid, Spain; 13 IDIPHISA Research Institute, Madrid, Spain; 14 Universidad Autónoma de Madrid, Madrid, Spain; 15 Endoscopy and Gastroenterology Unit, Pontchaillou University Hospital, Rennes, France; 16 Department of Gastroenterology and Endoscopy, Hôpital Edouard Herriot, Hospices Civils de Lyon, Lyon, France; 17 Gastroenterology and Endoscopy Unit, Dupuytren University Hospital, Limoges, France; 18 Department of Gastroenterology and Digestive, Endoscopy Unit, University Hospital of Dijon, Dijon, France; 19 Department of Gastroenterology, Nancy Regional University Hospital Center, Nancy, France; 20 Department of Gastroenterology, Hospital Universitario HM Montepríncipe, HM Hospitales, Madrid, Spain; 21 Department of Clinical Sciences, Universidad San Pablo-CEU, CEU Universities, Madrid, Spain; 22 Gastroenterology Unit, Fondation Hopital Saint Joseph, Paris, France; 23 Gastroenterology Unit, Assistance Publique – Hôpitaux de Paris (AP-HP), Paris, France; 24 Paris Est Creteil University (UPEC), Paris, France; 25 Department of Gastroenterology, Henri Mondor Hospital, Paris, France; 26 Gastroenterology, Hepatology and Digestive Oncology Department, Haut-Lévêque Hospital, Bordeaux, France; 27 Digestive System and Endoscopy Unit, Hospital Vithas Xanit, Benalmadena, Málaga, Spain; 28 Gastroenterology, Hospital Quirónsalud Málaga, Málaga, Spain; 29 Gastroenterology and Hepatology Department, Clinical and Translational Research in Digestive Diseases, Valdecilla Research Institute (IDIVAL), Santander, Spain; 30 Marqués de Valdecilla University Hospital, Santander, Spain; 31 Hepatogastroenterology, Hôpital Cochin, Asisstance Publique-Hôpitaux de Paris (AP-HP), Paris, France; 32 Department of Gastroenterology and Hepatology, Clinique CHC MontLégia, Liège, Belgium; 33 Therapeutic Digestive Endoscopy, CHC Liège, Liège, Belgium; 34 Department of Gastroenterology and Endoscopy, Clinique de l’Anjou, Angers, France; 35 Gastroenterology and Hepathology Department, CHRU de Nancy – Hôpitaux de Brabois, Nancy, France; 36 Department of Gastroenterology, Clinique Jules Verne, Nantes, France; 37 Gastroenterology Department, Hospital Clínico Universitario Lozano Blesa, Zaragoza, Spain; 38 Instituto de Investigación Sanitaria (IIS) Aragón, Zaragoza, Spain; 39 Centro de Investigación Biomédica en Red de Enfermedades Hepáticas y Digestivas (CIBEREHD), Madrid, Spain; 40 Gastroenterology, GI Endoscopy Unit, Hospital Universitari Germans Trias i Pujol, Badalona, Spain; 41 Gastroenterology, Hospital de Poniente de Almeria, El Ejido, Spain; 42 Hospital General Universitario de Ciudad Real, Ciudad Real, Spain; 43 Gastroenterology, Hospital Universitario Nuestra Señora de Candelaria, Santa Cruz de Tenerife, Spain; 44 General and Digestive Surgery, Hospital Universitario Nuestra Señora de Candelaria, Santa Cruz de Tenerife, Spain; 45 Department of Gastroenterology and Hepatology, Hospital Universitario Ramón y Cajal, Madrid, Spain; 46 Instituto Ramón y Cajal de Investigación Sanitaria (IRYCIS), Madrid, Spain; 47 Department of Gastroenterology and Hepatology, Hospital General Universitario Gregorio Marañón, Madrid, Spain; 48 Endoscopy Unit, Gastroenterology Department, Hospital Clínic de Barcelona, Barcelona, Spain; 49 Instituto de Investigaciones Biomédicas August Pi i Sunyer (IDIBAPS), Barcelona, Spain; 50 Departament de Medicina, Facultat de Medicina i Ciències de la Salut, Universitat de Barcelona (UB), Barcelona, Spain; 51 Endoscopy Unit, Department of Digestive Diseases, Hospital Costa del Sol, Marbella, Spain; 52 Department of Gastroenterology, Donostia University Hospital, San Sebastian, Spain; 53 Department of Endoscopy and Gastroenterology, University Hospital Centre Rennes, Rennes, France

## Abstract

**Background** The Limoges Bleeding Score estimates an individual’s risk of clinically significant delayed bleeding (CSDB) after colorectal endoscopic submucosal dissection (ESD). We aimed to validate and update this model in a Western setting.

**Methods** Procedural data and complications were prospectively recorded in French–Belgian (FECCO) and Spanish cohorts. The Limoges score was externally validated. A revised Western score was derived. Score performance was determined by discrimination and calibration. Internal validation was performed using bootstrapping and leave-one-out cross-validation. The score’s performance was independently assessed in both cohorts.

**Results** 4767 ESDs were included: 33.7 % rectal and 37.7 % proximal; median lesion size 50 mm; mean patient age 68.1 years; American Society of Anesthesiologists (ASA) score I–II 72.9 %; anticoagulants 10.9 %, and antiplatelets 17.0 %. CSDB prevalence was 6.8 %. The performance of the Limoges score was modest. A new score called DEBE (Delayed Bleeding ESD) was developed: age ≥ 75 years (2 points), lesion size ≥ 50 mm (5 points), ASA classification III–IV (4 points), location in the rectum (2 points) or proximal colon (1 point), anticoagulants (7 points) and antiplatelets (3 points). The DEBE score ranged from 0 to 23 points and categorized the patients into two groups (low risk 3.9 %; medium-high risk 14.2 %). The score showed acceptable discrimination (area under the curve 0.712), adequate calibration, and consistent performance after internal validation.

**Conclusions** The DEBE score, based on seven preprocedural variables, allowed a personalized assessment of bleeding risk. It determined the individual CSDB risk, identified patients who would benefit from prophylactic treatment, and defined those who require monitoring after ESD.

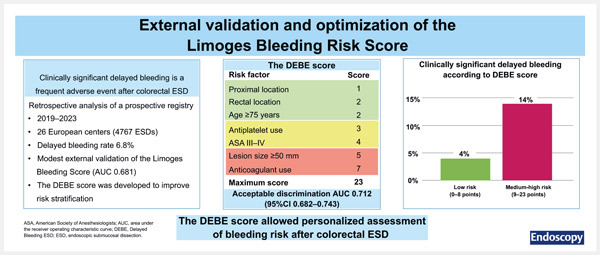

## Introduction

Endoscopic submucosal dissection (ESD) has become an essential technique for achieving en bloc and curative resection of large superficial colorectal lesions, resulting in low local recurrence rates 1 2 .

Clinically significant delayed bleeding (CSDB) is one of the most frequent adverse events of ESD. Its incidence varies across different cohorts, ranging from 1 % to 9 %, with no significant differences observed between Asian 3 4 and Western 5 6 populations. High CSDB rates were observed in both prospective and controlled studies. CSDB can pose a substantial burden; therefore, strategies aimed at minimizing this risk are highly warranted.

The available data on risk factors for CSDB after ESD are limited. A single-center study proposed a nomogram to estimate CSDB risk based on three variables: rectal location, lesion size, and procedure duration 7 . Similarly, a multicenter Korean study developed a risk score incorporating rectosigmoid location, lesion size ≥ 30 mm, and antiplatelet use 8 ; however, this score demonstrated low discriminative ability. Consequently, a new predictive model, the Limoges Bleeding Score, was developed. This score comprises five variables: age ≥ 75 years, size > 50 mm, comorbidity, rectal location, and use of anticoagulants/antiplatelets 6 .

The aim of this multicenter study was to further investigate risk factors for CSDB after colorectal ESD and to validate the Limoges score externally in a large Western cohort. We also aimed to update the model to improve risk stratification accuracy.

## Methods

### Study design

This study was a retrospective analysis based on prospectively collected data from 26 centers, encompassing two large European cohorts: French ESD Colorectal Cohort (FECCO NCT: 04592003, including one center from Belgium; September 2019 to May 2023; patients from a previously collected Limoges cohort were also included and used to create the Limoges score), and the cohort recruited by the Third Space Endoscopy Working Group of the Spanish Society of Gastrointestinal Endoscopy (GSEED) (Institutional Review Board approval number 14/384; January 2016 to May 2022). Patient demographics and pre- and periprocedural characteristics were gathered prospectively.

Ethical approvals were obtained from the ethics committees of all participating centers, in accordance with national regulations. Informed consent to enter the respective cohorts for data use and procedural participation was obtained from all patients following institutional protocols. A No Opposition to Data Use form was sent and signed by the patients.

A structured follow-up was carried out 30–60 days after ESD, through an in-person or telephone consultation, to review histopathological results and identify any delayed complications, including CSDB.

The therapeutic approach to CSDB was individualized, based on the attending gastroenterologist’s clinical judgment.

### Definitions

CSDB was defined as any significant bleeding event (hematochezia) occurring within 30 days after ESD that required emergency service stay, prolonged hospitalization, readmission, repeat endoscopy, angiographic embolization, surgery, or transfusion 6 .

Periprocedural perforation was identified as direct exposure of the peritoneal cavity due to a muscularis propria defect, corresponding to Sidney classification IV–V 9 . According to the European Society of Gastrointestinal Endoscopy (ESGE) guidelines 10 , histological outcomes were classified as follows: R0 resection if no neoplastic involvement was found at both vertical and lateral resection margins; curative resection in cases of R0 resection and the absence of high-risk features (submucosal invasion > 1000 µm, lymphovascular invasion, poor differentiation, and high-grade tumor budding).

The proximal colon was defined as the right colon, including the cecum, the ascending colon, and the hepatic flexure, but not including any of the transverse colon.

### Inclusion and exclusion criteria

All epithelial colorectal lesions treated by ESD during the specified inclusion period were prospectively and consecutively included in this study. Enrollment was determined at the time of ESD initiation.

To support the study’s integrity and reduce the risk of bias, centers had to contribute at least 50 patients to participate in the study. For patients with two or more lesions, only the largest lesion was included, and a colonoscopy was performed to confirm the source of bleeding in cases of CSDB. All lesions were taken into consideration if they were removed on different procedure days.

Lesions were excluded if they met any of the following criteria: failed ESD or resections using hybrid techniques that involved a snare; submucosal lesions, neuroendocrine tumors, or non-neoplastic lesions that were not serrated.

### ESD indications and procedural details

A total of 45 experienced endoscopists carried out the ESD procedures. While ESD indications may have varied between centers and over time, the majority of centers followed ESGE guidelines. Some centers adopted a more Japanese-inspired approach, indicating ESD for all large (> 25 mm) rectal laterally spreading tumors since 2013 and expanding to colonic lesions from 2016 6 .

The procedures were performed using single-channel, high-resolution endoscopes with the patient under deep sedation or general anesthesia. ESD was performed after a thorough optical assessment to verify the resectability of the lesion. The selection of lifting solutions, dissection knives, and dissection approaches, including traction methods, was tailored to operator preference and institutional protocols 11 . Electrosurgical units, types of currents, and prophylactic coagulation of visible vessels post-ESD were not standardized and depended on local practices. The majority of patients required an overnight stay following ESD, with prolonged stays designated for those who experienced adverse events.

Antithrombotic management, including adjustments to anticoagulant and antiplatelet therapy, was performed in accordance with ESGE guidelines 12 .

### Statistical analysis

Clinical characteristics were described using frequencies for categorical variables, and means (SDs) or medians (1st quartile [Q1]–3 rd quartile [Q3]) for continuous variables, depending on their distribution. To reduce potential bias from the “complete case” analysis, we performed single imputation by regression for the score variables within each dataset using the “mice” R package. Predictive mean matching (five donors) was performed for age and lesion size, whereas logistic regression was used for binary variables. Two-sided tests were used to evaluate all hypotheses. Comparisons between the groups were assessed using Student’s *t* tests or Mann–Whitney *U* tests for continuous variables, and chi-squared test or Fisher’s exact test for categorical variables, and a *P* value of < 0.05 was considered statistically significant. The incidence of CSDB was estimated using sample proportions with 95 %CIs.

The Limoges Bleeding Score was externally validated in both the Spanish and French–Belgian cohorts, excluding the Limoges patients. Model discrimination was described by the area under the receiver operating characteristic (ROC) curve (AUC) with 95 %CIs, and calibration by the Hosmer–Lemeshow goodness-of-fit test and calibration plots. We recalibrated the score using two approaches: Type 1, which re-estimates the intercept; and Type 2, which re-estimates the intercept and provides a new weight for the linear predictor of the logistic regression. We used a multivariable logistic regression model to derive a new model with these variables: age, polyp size, ASA class, polyp location, and anticoagulant or antiplatelet use. These factors were selected due to their established predictive efficacy 6 13 , their widespread clinical application, and their availability in datasets used for model development. To derive this new multivariable model, all available data were used, as recommended by Riley et al. 14 . The minimum required sample size was calculated using the pmsampsize function in R 14 , assuming an event prevalence of 6.8 % (observed in the total derivation sample), an expected c-statistic of at least 0.72, an acceptable difference in apparent and adjusted R-square of 0.05, and eight candidate parameters. This calculation resulted in a requirement for 1716 subjects (117 events), a number that was exceeded by our derivation cohort (n = 4767).

Model odds ratios (ORs) and 95 %CIs were calculated. We developed a risk score by assigning a weight to each predictor based on the β coefficients from the multivariable model. All variables included in the score were categorical, so no standardization or normalization was performed. The β coefficients of the multivariable regression model were defined as the log-odds for each category relative to the reference category. As previously described, we divided these β coefficients by the smallest β coefficient and rounded them to the nearest integer to calculate the score 15 16 17 . Discrimination and calibration were assessed. We validated the score internally using the resampling validation method for logistic models with 500 bootstrap re-samples. Then, we estimated the optimism-corrected ROC and calibration slope with their 95 %CIs. To allow for simple interpretation in a clinical setting, we divided the score into two categories using the global CSDB incidence in the cohort as the threshold. Leave-one-out cross-validation was performed as a second internal validation approach to calculate the score’s accuracy, using a probability threshold equal to the global CSDB incidence in the cohort. Finally, geographical validation was performed by evaluating calibration and discrimination using the Spanish GSEED cohort and the FECCO cohort, respectively, as suggested by Riley et al. 14 .

Statistical analyses were performed using the IBM Statistical Package for the Social Sciences version 25.0 (IBM Corp, Armonk, New York, USA) and R version 4.3.0 (The R Foundation for Statistical Computing, Vienna, Austria).

The study adhered to the Transparent Reporting of a multivariable prediction model for Individual Prognosis Or Diagnosis (TRIPOD) guidelines 18 .

## Results

### General characteristics of patients and lesions

**Fig. 1:**
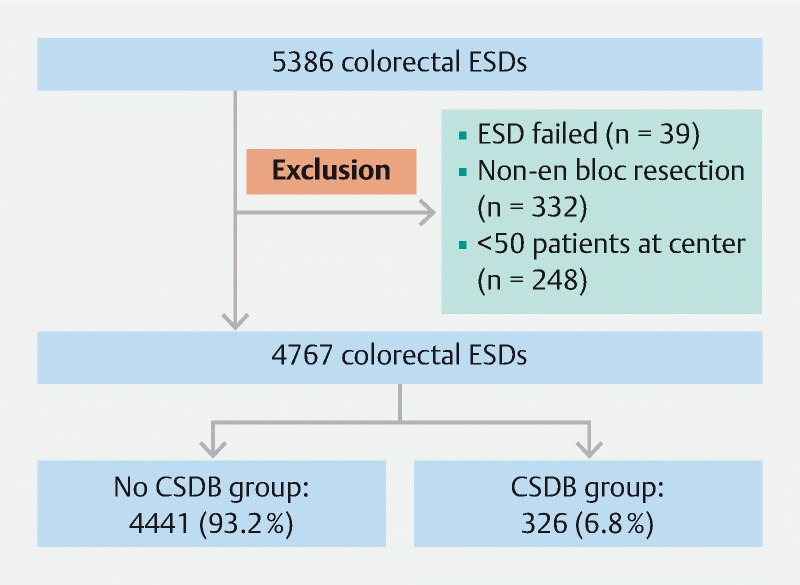
Flowchart of the study. CSDB, clinically significant delayed bleeding; ESD, endoscopic submucosal dissection.

A total of 5386 colorectal ESDs were recruited, but 39 were excluded due to technical failure, 332 due to non-en bloc resection, and 248 that were from centers with fewer than 50 cases. Thus, a total of 4767 consecutive colorectal ESDs were included ( Fig. 1 ). The median lesion size was 50.0 mm (Q1–Q3 36.0–65.0 mm) (see **Table 1 s** in the online-only Supplementary material).

**Table 1:** Baseline characteristics of the studied cohorts.

	FECCO cohort			GSEED cohort
	Limoges (N = 900), n (%)	Rest France + Belgium (N = 2736), n (%) ^1^	All France + Belgium (N = 3636), n (%) ^2^	Spain (GSEED cohort) (N = 1131), n (%) ^3^
Age group				
< 75 years	645 (71.1)	1972 (72.4)	2617 (72.2)	872 (77.3)
≥ 75 years	255 (28.3)	752 (27.6)	1007 (27.8)	256 (22.7)
Sex				
Female	408 (45.3)	1228 (44.9)	1636 (45.0)	449 (39.7)
Male	492 (54.7)	1508 (55.1)	2000 (55.0)	682 (60.3)
ASA classification				
I–II	576 (64.0)	2069 (77.0)	2645 (73.7)	794 (70.3)
III–IV	324 (36.0)	618 (23.0)	942 (26.3)	336 (29.7)
Size				
≤ 50 mm	394 (43.8)	1589 (58.2)	1983 (54.7)	688 (63.5)
> 50 mm	506 (56.2)	1139 (41.8)	1645 (45.3)	395 (36.5)
Anticoagulant therapy				
No	809 (89.9)	2374 (86.8)	3183 (87.5)	1065 (94.2)
Yes	91 (10.1)	362 (13.2)	453 (12.5)	66 (5.8)
Antiplatelet therapy				
No	730 (81.1)	2259 (82.6)	2989 (82.2)	966 (85.4)
Yes	170 (18.9)	477 (17.4)	647 (17.8)	165 (14.6)
Proximal location				
No	581 (64.6)	1691 (61.9)	2272 (62.5)	696 (61.5)
Yes	319 (35.4)	1042 (38.1)	1361 (37.5)	435 (38.5)
Rectum location				
No	542 (60.2)	1795 (65.7)	2337 (64.3)	820 (72.5)
Yes	358 (39.8)	938 (34.3)	1296 (35.7)	311 (27.5)
Data included in score development and validation				
Limoges score				
Development	☑	☒	☒	☒
External validation	☒	☑	☒	☑
DEBE score				
Development	☑	☑	☒	☑
Internal validation	☒	☒	☑	☑

ASA, American Society of Anesthesiologists; DEBE, Delayed Bleeding ESD; ESD, endoscopic submucosal dissection; FECCO, French ESD Colorectal Cohort; GSEED, Third Space Endoscopy Working Group of the Spanish Society of Gastrointestinal Endoscopy.

^1^ Age group N = 2724; ASA classification N = 2687; size N = 2728; location N = 2733. ^2^ Age group N = 3624; ASA classification N = 3587; size N = 3628; location N = 3633. ^3^ Age group N = 1128; sex, anticoagulant and antiplatelet therapy, location N = 1131; ASA classification N = 1130; size N = 1083.

### ESD characteristics

The median specimen surface area was 1484.4 mm ^2^ (Q1–Q3 824.7–2591.8). The median procedure duration was 69.0 minutes (Q1–Q3 40.0–120.0 minutes), with a mean procedure speed of 29.3 (SD 22.96) mm ^2^ /min. The main dissection current used was Swift coagulation (61.2 %). The resection was R0 in 4365 cases (91.6 %). Adrenaline was added in 909 cases (23.5 %), and a hemostatic agent was used in 38 cases (1.0 %). After ESD, 1535 defects (32.2 %) were closed with clips.

### Adverse events

CSDB occurred in 326 patients (6.8 %; 95 %CI 6.15 to 7.6), after a median of 2 days (Q1–Q3 1–9 days). In 77 cases (1.6 %), blood transfusions were necessary. Intraprocedural and delayed perforation occurred in 420 patients (10.9 %) and 62 patients (1.3 %), respectively. Complications led to prolonged hospitalization in 495 patients (10.4 %), and this was due to CSBD in 241 patients (5 %). Emergency attendance after discharge was required for 142 patients (3.7 %), and this was due to CSDB in 115 patients (2.4 %). Surgery due to complications was needed in 23 patients (0.6 %), and this was due to CSBD in 3 patients (0.06 %). Three patients (0.1 %) died due to procedure-related complications.

### Performance of the Limoges Bleeding Score model

The Limoges score model was first applied to the FECCO cohort, excluding Limoges data ( Table 1 ), obtaining a modest discriminative ability with an AUC of 0.681 (95 %CI 0.643 to 0.719). In terms of calibration, the mean risk calculated using the score (5.8 %) differed from the observed incidence (7.2 %). Additionally, the weight that the score variables had in predicting CSDB with respect to the derivation cohort differed. Therefore, type 2 recalibration was performed. This recalibration remained suboptimal. The model was then applied to the Spanish GSEED cohort ( Table 1 ), obtaining an AUC of 0.675 (95 %CI 0.604 to 0.746), suggesting a limited discriminative ability. In terms of calibration, the mean risk calculated with the score (5.3 %) was comparable to the observed incidence (5.0 %). However, the weight that the score variables had in predicting CSDB differed based on the derivation cohort. Consequently, type 2 recalibration was necessary; however, it remained modest.

Although the Limoges model performance was suboptimal, it facilitated the development of a more generalizable, predictive model applicable to a broader range of contexts.

### Development of the DEBE model

A new model was derived using multivariable logistic regression. The final model identified the following factors as predictive: age ≥ 75 years (OR 1.29, 95 %CI 1.00 to 1.66; *P* = 0.047), anticoagulant therapy (OR 2.94, 95 %CI 2.18 to 3.94; *P* < 0.001), antiplatelet use (OR 1.68, 95 %CI 1.26 to 2.22; *P* < 0.001), rectum location (OR 1.45, 95 %CI 1.07 to 1.97; *P* = 0.017), proximal location (OR 1.18, 95 %CI 0.86 to 1.61; *P* = 0.303), ASA classification III or IV (OR 1.81, 95 %CI 1.38 to 2.36; *P* < 0.001), and lesion size ≥ 50 mm (OR 2.16, 95 %CI 1.68 to 2.80; *P* < 0.001). Regression coefficients for the predictors were converted into points on a risk score ( Table 2 ).

**Table 2:** Summary of the analysis of risk factors for CSDB after endoscopic submucosal dissection and the assigned score points.

Predictors	β	CI	*P*	Score
(Intercept)	–3.89	–4.22 to –3.58	< 0.001	
Age (≥ 75)	0.26	0.00 to 0.51	0.047	2
Size (≥ 50)	0.77	0.52 to 1.03	< 0.001	5
ASA (III–IV)	0.59	0.32 to 0.86	< 0.001	4
Location (proximal)	0.16	–0.14 to 0.48	0.303	1
Location (rectum)	0.37	0.07 to 0.68	0.017	2
Anticoagulants	1.08	0.78 to 1.37	< 0.001	7
Antiplatelets	0.52	0.23 to 0.80	< 0.001	3
Observations	4767			

ASA, American Society of Anesthesiologists; CSDB, clinically significant delayed bleeding.

**Fig. 2:**
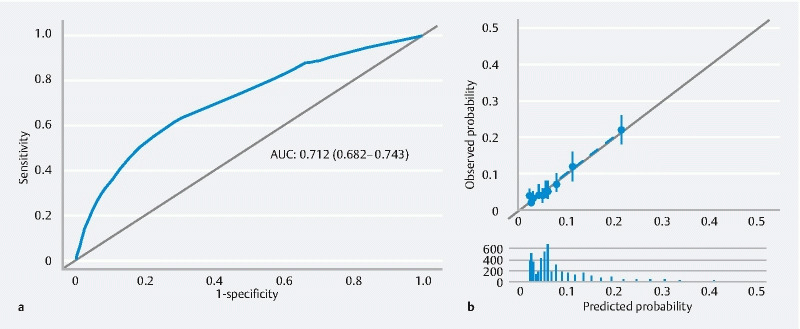
The new Delayed Bleeding ESD (DEBE) score. **a** Receiver operating characteristic curve. **b** Calibration plot. AUC, area under the receiver operating characteristic curve; ESD, endoscopic submucosal dissection.

The new Delayed Bleeding ESD (DEBE) score had an AUC of 0.712 (95 %CI 0.682 to 0.743), showing acceptable discrimination ( Fig. 2a ). The bias-corrected index after internal bootstrap validation was 0.713 (95 %CI 0.678 to 0.745). The calibration of the model was found to be satisfactory, as indicated by the calibration curves ( Fig. 2b ) and the Hosmer–Lemeshow test results (chi-squared 4.334; *P* = 0.83). The discrimination slope (the difference in average predictions for those with and without the outcome) was 0.06 (95 %CI 0.04 to 0.08).

Additionally, leave-one-out cross-validation was performed to calculate the accuracy of the score (measured as the percentage of corrected classified patients), obtaining a value of 72.6 %.

The CSDB probability score ranges from 0 (lowest risk) to 23 (medium-high risk). A cutoff point between 8 and 9, based on the average incidence of CSDB (6.8 %), categorized the patients into two risk groups (low risk 3.9 %; medium-high risk 14.2 %).

The following formula was used to calculate the individual CSDB risk for each patient:

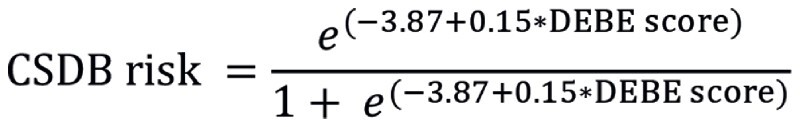



The probability of CSDB according to the score is listed in Table 3 .

**Table 3:** Probability of CSDB according to the new Delayed Bleeding ESD (DEBE) score.

Score	Patients	CSDB probability, %	CSDB patients, n (%)	Score range	CSDB patients/patients	CSDB probability, %	CSDB risk
0	377	2	9 (2.4)	0 to 8	132 /3399	3.9	LOW*
1	516	2.4	13 (2.5)				
2	369	2.8	11 (3.0)				
3	130	3.2	5 (3.8)				
4	166	3.7	3 (1.8)				
5	428	4.3	23 (5.4)				
6	544	5.0	25 (4.6)				
7	681	5.7	31 (4.6)				
8	188	6.6	12 (6.4)				
9	303	7.6	23 (7.6)	9 to 23	194/1368	14.2	MEDIUM-HIGH*
10	165	8.8	14 (8.5)				
11	154	10.1	18 (11.7)				
12	132	11.5	18 (13.6)				
13	149	13.2	18 (12.1)				
14	118	15.0	18 (15.3)				
15	72	17.1	13 (18.1)				
16	84	19.4	20 (23.8)				
17	27	21.9	4 (14.8)				
18	47	24.6	14 (29.8)				
19	38	27.5	11 (28.9)				
20	47	30.7	12 (25.5)				
21	13	34.0	4 (30.8)				
22	6	37.5	3 (50.0)				
23	13	41.2	4 (30.8)				

CSDB, clinically significant delayed bleeding; ESD, endoscopic submucosal dissection. * A cutoff point between 8 and 9, based on the average incidence of CSDB (6.8%), categorize the patients into two risk groups.

When applied to our population, the data revealed that 3399 patients (71.3 %) were below the average CSDB risk and 1368 (28.7 %) were above this risk threshold of 6.8 %.

### Performance of the DEBE model in the two European areas

Finally, the new DEBE score was studied separately in the two geographical areas, FECCO (France + Belgium) and GSEED (Spain) cohorts ( Table 1 ).

**Fig. 3:**
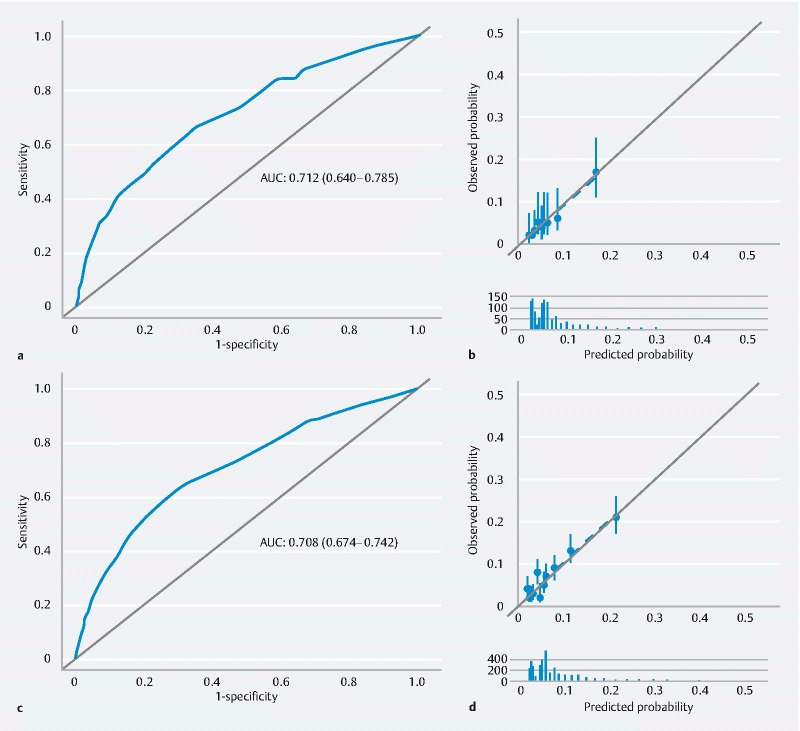
The new Delayed Bleeding ESD (DEBE) score applied to datasets from Spanish ( **a,b** ) and French–Belgian ( **c,d** ) cohorts. **a,c** Receiver operating characteristic curves. **b,d** Calibration plots. AUC, area under the receiver operating characteristic curve; ESD, endoscopic submucosal dissection.

In the Spanish (GSEED) dataset (n = 1131), the mean risk calculated with the new DEBE score (5.62 %) was close to the observed incidence (5.04 %; 95 %CI 3.87 to 6.52). The new DEBE score was applied to the Spanish cohort, resulting in acceptable discriminatory capacity (AUC 0.712; 95 %CI 0.640 to 0.785) ( Fig. 3a ). Calibration ( Fig. 3b ) and predictive accuracy (Brier score 0.046; 95 %CI 0.04 to 0.06) were found to be satisfactory.

In the FECCO dataset (n = 3636), the mean risk calculated with the new DEBE score (7.0 %) was consistent with the observed incidence (7.4 %; 95 %CI 6.58 to 8.31). The new DEBE score was applied to this dataset, resulting in an acceptable discrimination ability (AUC 0.708; 95 %CI 0.674 to 0.742) ( Fig. 3c ). Calibration ( Fig. 3 d ) and predictive accuracy (Brier score 0.065; 95 %CI 0.06 to 0.07) showed adequate reliability.

## Discussion

In this large cohort study, the incidence of CSDB was 6.8 % (326/4767), which aligns with other series 1 6 19 . The Limoges score 6 demonstrated limited discrimination in predicting CSDB in both the Spanish and French–Belgian cohorts included in our study. Therefore, we developed an updated model, the DEBE score, based on the same five predictor factors (anticoagulants/antiplatelets, lesion size, ASA classification, age, and location), allowing for a personalized assessment of the bleeding risk in a Western context.

The DEBE score assigns greater weight to anticoagulant use (7 points), considering it different from antiplatelet use (3 points). Additionally, lesions ≥ 50 mm have been given increased significance (5 points). Proximal location has been added, although its impact on bleeding risk is less pronounced (1 point).

The DEBE score demonstrated an AUC of 0.712, indicating acceptable discrimination. Notably, it exhibited excellent calibration and discrimination properties, along with stability across both cohorts – 0.712 (GSEED cohort) and 0.708 (FECCO cohort) – without the necessity for recalibration. The higher AUC achieved for the Limoges score during internal validation (0.751) was primarily attributable to the higher prevalence of CSDB within the sample (8.0 %) 6 . However, its performance was unsatisfactory when tested on external cohorts in the current study (AUC FECCO 0.681, GSEED 0.675).

The most relevant risk factor identified was anticoagulant use (10.9 %), which had an incidence of CSDB of 18.7 % and an OR of 2.94. This finding is consistent with previous studies 3 20 21 and reflects the increasing use of anticoagulants worldwide. The DEBE score has the potential to personalize anticoagulation management in high-risk patients. However, it is important to exercise caution, as delayed resumption of anticoagulants has been associated with an increased risk of thromboembolic events 21 .

A lesion size ≥ 50 mm was the second most impactful risk factor (OR 2.16). Given the established correlation between larger lesions and a greater number of injured or sectioned submucosal vessels, lesion size is a significant and expected risk factor for delayed bleeding in both ESD 6 7 8 22 and endoscopic mucosal resection (EMR) cohorts 16 23 . The median lesion size in our study was 50 mm (60 mm in the CSDB group), a value that is notably different from other series, which included smaller lesions and had a lower incidence of CSDB 8 24 .

Rectal location has been identified as a significant risk factor for CSDB following colorectal ESD 6 7 13 19 22 . Proximal location is a well-established risk factor for delayed bleeding after colonic EMR 16 23 . However, this finding is not consistent across ESD studies. Two retrospective studies and our previous work reported an increased risk of CSDB after ESD in proximal lesions 13 20 25 . This variable was included in the DEBE score based on clinical relevance and its contribution to the score’s performance, rather than its statistical significance. The reasons for higher CSDB risk in rectal lesions after ESD and in the proximal colon after EMR remain unclear but they may be related to vascular anatomical differences 26 . Both rectal and proximal locations were included in the DEBE score (2 and 1 points, respectively).

The DEBE score is divided into two groups based on the average incidence of CSDB (cutoff point): low risk (< 9 points, 3.9 % bleeding rate) and medium-high risk (9–23 points, 14.2 % bleeding rate). Nevertheless, we conducted a more comprehensive analysis of risk factors, and the DEBE score enabled the classification of bleeding risk into 24 categories, providing the highest accurate individual risk stratification compared with other scores 16 23 27 . In this context, preventive strategies such as prophylactic clipping, advanced closure methods 28 , and hemostatic gels 29 may be considered.

It is of clinical interest to reduce the morbidity of the high-risk cohort because of the significant economic and ecological burden of CSDB, including hospital stays – sometimes in intensive care units – new procedures, and additional treatments. Research has been conducted on various methods for reducing bleeding. Prophylactic clipping in large right-sided lesions is now recommended after EMR 30 . However, the CSDB risk factors after EMR and ESD are not equivalent, and there is less evidence available regarding the efficacy of clip closure after ESD. Therefore, no specific recommendations can be made in this setting. A retrospective study reported a significant reduction in CSDB rates after colorectal ESD when complete clip closure was performed in patients on direct oral anticoagulants (10.8 % vs. 5.2 %) and warfarin (17.1 % vs. 6.1 %), with the risk reduction being particularly relevant for right-sided lesions 20 . Another observational study indicated a 4.9 % bleeding risk in anticoagulated patients when clip closure was achieved 24 . Other classic studies conducted in Japan recognized the effect of clips 4 , as did a recent study 31 . However, other studies, including one recently published by our group 32 , have not reported reduced CSDB rates after clipping ESD defects 33 34 . In the current study, 32.2 % of the patients underwent complete clip closure, with no reduction in the CSDB incidence. Despite this, the study was not designed to evaluate the effectiveness of defect closure in preventing CSDB, and our data are insufficient to provide conclusive evidence to address this knowledge gap. Consequently, our model’s ability to identify high-risk patients who may benefit from prophylactic strategies could inform the design of clinical trials specifically aimed at reducing CSDB after colorectal ESD.

The key strengths of this study lie in its substantial sample size and multicenter design. These elements bolster the external validity of our model. The geographical validation of the DEBE score in two sub-cohorts representing diverse European regions further substantiates the conclusions drawn from the study 14 . It is noteworthy that we included exclusively successful colorectal ESD cases, excluding any snare intervention, which is a notable distinction from other studies 8 .

With regard to the study limitations, it is essential to exercise caution, as the updated score has not yet undergone external validation by other independent working groups. Nonetheless, the multicenter design and the implementation of different validation methods (bootstrapping, leave-one-out analysis, and geographical validation) suggest considerable potential for applicability in different scenarios.

Longer procedure times have been identified as an independent risk factor for CSDB 5 7 35 . However, this variable was not incorporated into the DEBE score, as it was designed to be a straightforward model, based solely on preprocedural data to identify high-risk patients before ESD.

Unlike other studies 35 , we did not perform direct comparisons between different anticoagulants, antiplatelets, or combinations. Including too many predictors in the score could reduce the precision and reliability of the regression coefficient estimates. Furthermore, the extended inclusion period may be viewed as a potential source of bias due to the possibility of evolving practices. However, no new hemostasis tools altered practice during this period, and the French cohort, which included a short period (4 years), reproduced the favorable results of the score.

In conclusion, the use of anticoagulants or antiplatelet agents, lesion size ≥ 50 mm, severe comorbidity, age ≥ 75 years, and lesion location in the rectum or proximal colon, were identified as risk factors for CSDB following colorectal ESD. Integrating these factors into the DEBE score allowed for the accurate prediction of bleeding risk prior to ESD with good calibration and discrimination properties. The score demonstrated consistent performance across two distinct European cohorts. This model can assist endoscopists in predicting bleeding risk, guiding decisions regarding prophylactic interventions when necessary, and facilitating the design of clinical trials to evaluate the efficacy of bleeding prevention strategies in selected high-risk patients.

## Data transparency statement

Data, analytic methods, and study materials will not be made available to other researchers. Individual participant data will not be shared. Anonymised, aggregated data will be shared with partners for research purposes only. The data derived from this research that supports our findings will be made available to interested parties upon reasonable request by the corresponding author.

## Supplementary Material

Supplementary material Zusatzmaterial
