# Supplementary material for: External validation and optimization of the Limoges score to improve the delayed bleeding risk prediction after colorectal endoscopic submucosal dissection: the Delayed Bleeding ESD (DEBE) score
Source: Endoscopy. 2026 Mar 23;58(7):741–52. doi: 10.1055/a-2814-4950 (PMC13286675; doi:10.1055/a-2814-4950)
Supplement: Supplementary file 1 — Supplementary material [file 26038supmat_10-1055-a-2814-4950.pdf]

Supplementary material

External validation and optimization of the Limoges score to improve the delayed bleeding risk prediction after colorectal endoscopic submucosal dissection: the Delayed Bleeding ESD (DEBE) score

Eduardo Albéniz, Sheyla Montori, Mónica Enguita-Germán, José Carlos Marín Gabriel, Alberto Herreros de Tejada, Timothée Wallenhorst, Jérôme Rivory, Romain Legros, Thibault Degand, Jean-Baptiste Chevaux, Felipe Ramos-Zabala, Yann Le Baleur, Florian Rostain, Arthur Berger, Pedro J. Rosón, Álvaro Terán, Maximilien Barret, Philipe Leclercq, Edouard Chabrun, Marion Schaeffer, Bertrand Brieau, Safia Boukechiche, Gonzalo Hijos-Mallada, Mathieu Pioche, Jérémie Jacques on behalf of the Colorectal ESD Consortium Working Group

Table 1s Patient and lesion characteristics by delayed bleeding.

Variable	Categories	n total (%) 4767 (100.0)	No CSDB (%) 4441 (93.2)	CSDB (%) 326 (6.8)	p-value
**Demographic characteristics**					
Sex	Male	**2682 (56.3)**	2478 (92.4)	204 (7.6)	0.020*
	Female	**2085 (43.7)**	1963 (94.1)	122 (5.9)	
Age, years	Mean (SD)	**68.1 (10.3)**	68.0 (10.0)	71.6 (10.2)	<0.001*
Age group	<75	**3489 (73.4)**	3314 (94.6)	191 (5.4)	<0.001*
	>=75	**1263 (26.6)**	1127 (89.3)	135 (10.7)	
**Clinical characteristics**					
ASA classification	I-II	**3439 (72.9)**	3313 (95.3)	164 (4.7)	0.011*
	III-IV	**1278 (27.1)**	1128 (87.4)	162 (12.6)	
Antiplatelet/anticoagulant therapy	No	**3500 (73.4)**	3342 (95.5)	158 (4.5)	<0.001*
	Yes	**1267 (26.6)**	1099 (86.7)	168 (13.3)	
Anticoagulant therapy	No	**4248 (89.1)**	4019 (94.6)	229 (5.4)	<0.001*
	Yes	**519 (10.9)**	422 (81.3)	97 (18.7)	
Antiplatelet therapy	No	**3955 (83.0)**	3718 (94.0)	237 (6.0)	<0.001*
	Yes	**812 (17.0)**	723 (89.0)	89 (11.0)	
**Lesion characteristics**					
Location	Distal	**1361 (28.6)**	1289 (94.7)	72 (5.3)	0.001*
	Proximal (without transverse)	**1796 (37.7)**	1684 (93.7)	114 (6.3)	
	Rectum	**1607 (33.7)**	1468 (91.3)	140 (8.7)	
Rectum location	No	**3157 (66.3)**	2973 (94.1)	186 (5.9)	<0.001*
	Yes	**1607 (33.7)**	1468 (91.3)	140 (8.7)	
Lesion size, mm	Median (P25 - P75)	**50.0 (36.0 - 65.0)**	50.0 (35.0 - 65.0)	60.0 (45.0 - 80.0)	<0.001*
Size 50 mm	<=50	**2671 (56.7)**	2574 (95.3)	128 (4.7)	<0.001*
	>50	**2040 (43.3)**	1867 (90.4)	198 (9.6)	
Paris classification	Protuding	**2034 (42.7)**	1870 (91.9)	164 (8.1)	<0.001*
	Non-protuding	**2441 (51.2)**	2291 (93.9)	150 (6.1)	
	Not classified	**292 (6.1)**	280 (95.9)	12 (4.1)	
JNET	JNET I	**112 (4.0)**	109 (97.3)	3 (2.7)	0.160
	JNET IIA	**1607 (58.1)**	1501 (93.4)	106 (6.6)	
	JNET IIB	**986 (35.6)**	917 (93.0)	69 (7.0)	
	JNET III	**62 (2.2)**	55 (88.7)	7 (11.3)	
Pathological analysis	Normal	**13 (0.3)**	12 (92.3)	1 (7.7)	0.116
	LGD	**1800 (37.8)**	1697 (94.3)	103 (5.7)	
	HGD	**2265 (47.5)**	2091 (92.3)	174 (7.7)	
	Superficial sm cancer (<1000)	**184 (3.9)**	174 (94.6)	10 (5.4)	

	Deep sm cancer (>1000)	**239 (5.0)**	217 (90.8)	22 (9.2)	
	SSL	**127 (2.7)**	121 (95.3)	6 (4.7)	
	T2	**37 (0.8)**	32 (86.5)	5 (13.5)	
	Others	**2 (0.0)**	2 (100.0)	0 (0.0)	
Fibrosis	F0	**1886 (43.1)**	1780 (94.4)	106 (5.6)	
	F1	**1314 (30.0)**	1226 (93.3)	88 (6.7)	0.035*
	F2	**1175 (26.9)**	1081 (92.0)	94 (8.0)	

CSDB, clinically significant delayed bleeding; SD, Standard Deviation; ASA, American Society of Anesthesiologists; JNET, Japan NBI Expert Team; LGD, low grade dysplasia; HGD, high grade dysplasia; P25 - P75, first and third quartiles. The information in the "Total" column is presented as a frequency (%) by column, whereas the information in the "No CSDB" and "CSDB" columns is presented as a frequency (%) by row. * $p < 0.05$
